# Platelet function-guided modification in antiplatelet therapy after acute ischemic stroke is associated with clinical outcomes in patients with aspirin nonresponse

**DOI:** 10.18632/oncotarget.22293

**Published:** 2017-11-07

**Authors:** Xingyang Yi, Jing Lin, Chun Wang, Ruyue Huang, Zhao Han, Jie Li

**Affiliations:** ^1^ Department of Neurology, People’s Hospital of Deyang City, Deyang 618000, Sichuan, China; ^2^ Department of Neurology, The Third Affiliated Hospital of Wenzhou Medical University, Wenzhou 325200, Zhejiang, China; ^3^ Department of Neurology, The Second Affiliated Hospital and Yuying Children’s Hospital of Wenzhou Medical University, Wenzhou 325027, Zhejiang, China

**Keywords:** aspirin, ischemic stroke, resistance, platelet function testing, nonresponse

## Abstract

**Purpose:**

To investigate the association of clinical outcomes with platelet function-guided modification in antiplatelet therapy in patients with ischemic stroke.

**Results:**

Among 812 patients, 223 patients had aspirin nonresponse, 204 patients was modified in antiplatelet therapy after platelet function testing. Mean follow-up period was 4.8 ± 1.7 years (ranged from 1 to 6.4 years). The incidence rates of ischemic events, death, or bleeding events were not significantly different between the patients with and without antiplatelet therapy modification. However, in patients with aspirin nonresponse, antiplatelet therapy modification was associated with decreased ischemic events (hazard ratio, 0.67; 95% confidence interval [CI], 0.62–0.97; *P* = 0.01) and ischemic stroke (hazard ratio, 0.70; 95% CI, 0.63–0.98; *P* = 0.03) compared with no modification in antiplatelet therapy.

**Conclusions:**

In patients with aspirin nonresponse, platelet function-guided modification in antiplatelet therapy after an ischemic stroke was associated with significantly lower rate of ischemic events. The platelet function testing may be useful to guide antiplatelet therapy modification.

**Methods:**

This is a retrospective, multicentre study. From August 2010 to December 2014, 812 patients with ischemic stroke underwent platelet function testing using platelet aggregation. Antiplatelet therapy modification was defined as any change in antiplatelet therapy after testing, including increasing aspirin dosage, adding an additional antiplatelet agent to aspirin, or switching to a more potent antiplatelet agent. The primary outcome was ischemic events. Secondary outcomes included death and bleeding events. Clinical outcomes were compared between patients with and without antiplatelet therapy modification using univariate and propensity score-adjusted analyses.

## INTRODUCTION

Stroke is a leading cause of mortality and disability [1]. In China, the age-standardized incidence rates per 100,000 person years of overall first-ever stroke were 135.0–270.0, the death rate of stroke was 11.4–15.4% during 1 year poststroke [2, 3]. There are approximately 3 million new stroke cases every year in China, with ischemic stroke accounting for 78.9% of all stroke [2]. The risk of recurrent stroke is very high after ischemic stroke [4]. After an ischemic stroke or transient ischemic attack (TIA) of arterial origin, antiplatelet therapy, such as aspirin or clopidogrel is currently recommended to reduce the risk of recurrent ischemic events [5, 6]. However, the response to aspirin is variable [7, 8]. The prevalence of aspirin nonresponse ranges from 5% to 60% [9, 10]. Our previous studies showed that nonresponse to aspirin in patients with ischemic stroke is associated with an increased risk of recurrence ischemic stroke (RIS) and worse functional status [8, 11].

Despite aspirin nonresponse signifying a risk factor for adverse events, there are no widely accepted standardized treatment recommendations for these patients. Increasing the dose of aspirin might reduce the rate of aspirin nonresponse, and prevent occurrence of vascular events [12, 13], but this may increase the risk of a hemorrhagic event [14]. Adding an additional antiplatelet agent combination therapy may be useful. The Clopidogrel in High-Risk Patients with Acute Nondisabling Cerebrovascular Events (CHANCE) trial showed that the combination of clopidogrel and aspirin for the first 21 days is superior to aspirin alone for reducing the risk of stroke in the first 90 days and does not increase the risk of hemorrhage in patients with TIA or minor stroke [15]. However, the MATCH (Management of Atherothrombosis with Clopidogrel in High-risk Patients with Recent Transient Ischaemic Attack or Ischaemic Stroke) trial found that long-term combination of clopidogrel and aspirin was not more effective than clopidogrel alone in preventing recurrent ischemic events, and the risk of life-threatening or major bleeding is increased [16]. Substitution of aspirin with other antiplatelet drugs is thought to offset the effect of antiplatelet drug resistance, and may help prevent the occurrence of vascular events [12]. In a trial of patients receiving coronary stents showed no significant improvements in clinical outcomes with platelet-function monitoring and treatment adjustment for coronary stenting [17]. Improvement in clinical outcomes by intensifying antiplatelet therapy has also not been demonstrated in patients with ischemic stroke or TIA [18, 19]. A retrospective study showed that platelet function-guided modification in antiplatelet therapy after an ischemic stroke or TIA was associated with significantly increased rates of death, ischemic events, or bleeding events [14]. However, Alberts reported that modification in antiplatelet therapy according to platelet function testing was reasonable [20]. Researchers of the latter studies maintain, however, that more data are required before any firm conclusion can be drawn.

The aim of the present study was to investigate the clinical efficacy and safety of platelet function-guided modification in antiplatelet therapy in patients with acute ischemic stroke. This may be useful to guide the precise treatment of antiplatelet drugs, and develop more effective drugs to prevent recurrent ischemic events after ischemic stroke.

## RESULTS

### Characteristics of patients

All patients were administered 200 mg aspirin per day for 14 days after the onset of stroke and 100 mg/day thereafter. Among the 812 patients, 223 patients (27.5%) had aspirin nonresponse according to platelet function testing. Table 1 compares the parameters between patients with and without aspirin nonresponse. The rate of diabetes mellitus and the level of fasting glucose were higher in patients with aspirin nonresponse than in those with AS (*P* < 0.001). There was no significant difference in other risk factors between the two groups.

**Table 1:** Baseline characteristics of patients with and without aspirin nonresponse

Parameter	Aspirin nonresponser *n* = 223	Aspirin sensitivity *n* = 589	*P* value^*^
Age (years)	70.7 ± 12.8	70.1 ± 11.4	0.76
Men (*n*, %)	107 (48.0)	317 (53.8)	0.24
Body mass index (kg/m^2^)	24.6 ± 3.4	24.1 ± 3.5	0.91
Current smoking (*n*, %)	63 (28.3)	172 (29.2)	0.98
Hypertension (*n*, %)	166 (74.5)	421 (71.5)	0.43
Diabetes mellitus (*n*, %)	79 (35.4)	93 (15.8)	<0.001
Previous MI (*n*, %)	6 (2.7)	10 (1.7)	0.42
NIHSS score at enrollment	5.9 ± 1.8	5.8 ± 1.9	0.89
Hyperlipidemia (*n*, %)	188 (84.3)	478 (81.2)	0.52
Fasting glucose (mmol/L)	7.2 ± 2.3	6.5 ± 2.5	<0.001
Platelet count (×10^9^/L)	193.2 ±28.8	196.5 ± 30.7	0.87
Stroke subtype Atherothrombotic (*n*, %) Small artery disease (*n*, %)	139 (62.3) 84 (37.7)	350 (59.4) 239 (40.6)	0.51 051
Previous treatment (*n*, %) Antihypertensive drugs Hypoglycemic drugs Statins Aspirin	97 (43.5) 52 (23.3) 35 (15.7) 50 (22.4)	263 (44.7) 92 (15.6) 99 (16.8) 141 (23.9)	0.88 0.014 0.74 0.73

MI, myocardial infarction; NIHSS, National Institutes of Health Stroke Scale.

### Antiplatelet therapy modification

Among the 812 patients, 204 patients (25.1%) were modified in antiplatelet therapy after platelet function testing (154 in aspirin nonresponse group, 50 in AS group). 50 patients with AS received modification in antiplatelet theraphy, because side effects of aspirin, such as allergic to aspirin (*n =* 1), asthma (*n =* 2), gastrointestinal bleeding (*n =* 8), hematuria (*n =* 5), skin or mucosal bleeding (*n =* 16), or severe nausea and vomiting (*n =* 18).

Baseline characteristics for the patients with (*n =* 204) and without (*n =* 608) ATM were shown in Table 2. Patients who underwent ATM were older, had higher platelet aggregation with AA or ADP compared with patients without ATM. Aspirin nonresponse was significantly higher in patients with ATM compared with patients without any modification.

**Table 2:** Baseline characteristics of patients with and without antiplatelet therapy modification

	Antiplatelet Therapy Modification		*P* value
	Yes (*n* = 204)	No (*n* = 608)	
Age (years)	71.8 ± 11.6	67.1 ± 13.6	<0.001
Men (*n*, %)	106 (51.9)	318 (52.3)	0.99
Diabetes mellitus (*n*, %)	50 (24.5)	122 (20.1)	0.18
Hypertension (*n*, %)	152 (74.5)	435 (71.5)	0.42
Body mass index (kg/m^2^)	24.5 ± 5.2	23.9 ± 4.9	0.15
Current smoker (*n*, %)	68 (33.3)	167 (27.5)	0.12
Previous MI (*n*, %)	6 (2.9)	10 (1.6)	0.26
Hyperlipidemia (*n*, %)	171(83.8)	495 (81.4)	0.43
Admission NIHSS	5.93 ± 1.8	5.86 ± 1.9	0.64
Stroke subtype Atherothrombotic (*n*, %) Small artery disease (*n*, %)	127 (62.3) 77 (37.7)	362 (59.5) 246 (40.5)	0.49 0.49
Previous treatment (*n*, %) Antihypertensive drugs Hypoglycemic drugs Statins Aspirin	89 (43.6) 39 (19.1) 32 (15.7) 46 (22.5)	271 (44.6) 105 (17.3) 102 (16.8) 145 (23.8)	0.83 0.56 0.72 0.71
In-hospital treatment (*n*, %) Antihypertensive drugs Hypoglycemic drugs Statins Thrombolysis	170 (83.3) 65 (31.9) 200 (98.0) 4 (2.0)	486 (79.9) 169 (27.8) 598 (98.4) 16 (2.6)	0.33 0.28 0.76 0.61
Platelet function testing			
Aggregation with AA, %	26.8 ± 10.2	20.1 ± 8.7	<0.001
Aggregation with ADP, %	58.4 ± 18.6	47.6 ± 16.4	<0.001
Aspirin nonresponse	154 (75.5)	69 (11.3)	<0.001
Aspirin sensitivity	50 (24.5)	539 (88.7)	<0.001
Follow-up period (years)	4.7 ± 1.6	4.8 ± 1.7	0.42

MI, myocardial infarction; NIHSS, national institutes of health stroke scale; AA, arachidonic acid; ADP, adenosine diphosphate.

The ATM after platelet function testing are shown in Table 3. Changing from aspirin to clopidogrel (*n =* 126, 61.8%) was the most common modifications. Clopidogrel was added to aspirin in 37 patients (18.1%). 23 patients (11.3%) were changed from aspirin to cilostazol. 18 patients (8.8%) were increased the aspirin dosage.

**Table 3:** Modification in antiplatelet therapy after platelet function testing

Modification in Antiplatelet Therapy	*n* = 204
Changed from aspirin to clopidogrel	126 (61.8%)
Changed from aspirin to cilostazol	23 (11.3%)
Increased aspirin	18 (8.8%)
Added clopidogrel to aspirin	37 (18.1%)

In aspirin nonresponders (*n =* 223), antiplatelet therapy was modified in 154 patients by changing from aspirin to clopidogrel (*n =* 97), adding clopidogrel to aspirin (*n =* 32), changing from aspirin to cilostazol (*n =* 15), increasing the aspirin dosage (*n =* 10). 69 aspirin non-responders did not receive ATM, because they were not willing to receive ATM.

### Clinical outcomes

Clinical follow-up was available for all patients with a mean follow-up period of 4.8 ± 1.7 years (ranged from 1 to 6.4 years). Ischemic events occurred in 181 (22.3%) patients (120 had ischemic stroke, 37 had TIA and 24 had MI). Bleeding events occurred in 83 (10.2%) patients. The incidence rates of is-chemic events, bleeding events, and death were not significantly different between the patients who underwent ATM compared with patients without modification (all *P* > 0.05, Table 4). There were also no significant differences in incidence rates of ischemic events and death among the different ATM (Supplementary Table 1). With regard to the patients in whom clopidogrel was added, the rate of bleeding was significantly higher than the patients without modification (27.0% [10/37] versus 9.9% [60/608], *P* < 0.001) or than the patients with modifications of changing from aspirin to clopidogrel or cilostazol (Supplementary Table 1). Retesting platelet function at 10 days after antiplatelet therapy modification was performed in 105 patients (51.5%). In patients with aspirin nonresponse, 76 % were responsive by adding clopidogrel, 52% were responsive by changing from aspirin to clopidogrel or cilostazol, and 41% were responsive by increasing the aspirin dosage.

**Table 4:** Clinical outcomes in patients with or without antiplatelet therapy modification

Variable	Antiplatelet Therapy Modification		*P* value
	Yes (*n* = 204)	No (*n* = 608)	
Ischemic events (*n*, %)	43 (21.1)	138 (22.7)	0.71
Ischemic stroke (*n*, %)	29 (14.2)	91 (15.0)	0.82
Transient ischemic attack (*n*, %)	8 (3.9)	29 (4.8)	0.68
Myocardial infarction (*n*, %)	6 (2.9)	18 (3.0)	0.99
Any bleeding event	23 (11.3)	60 (9.9)	0.61
GUSTO minor (*n*, %)	12 (5.9)	31 (5.1)	0.72
GUSTO moderate (*n*, %)	8 (3.9)	21 (3.5)	0.76
GUSTO severe (*n*, %) Gastrointestinal bleeding (n, %) Intracerebral hemorrhage (n, %)	3 (1.5) 12 (5.9) 2 (1.0)	8 (1.3) 41 (6.7) 7 (1.2)	0.94 0.72 0.98
Death (*n*, %)	7 (3.4)	19 (3.1)	0.84

GUSTO, Global Use of Strategies to Open Occluded Coronary Arteries.

**Figure 1:**
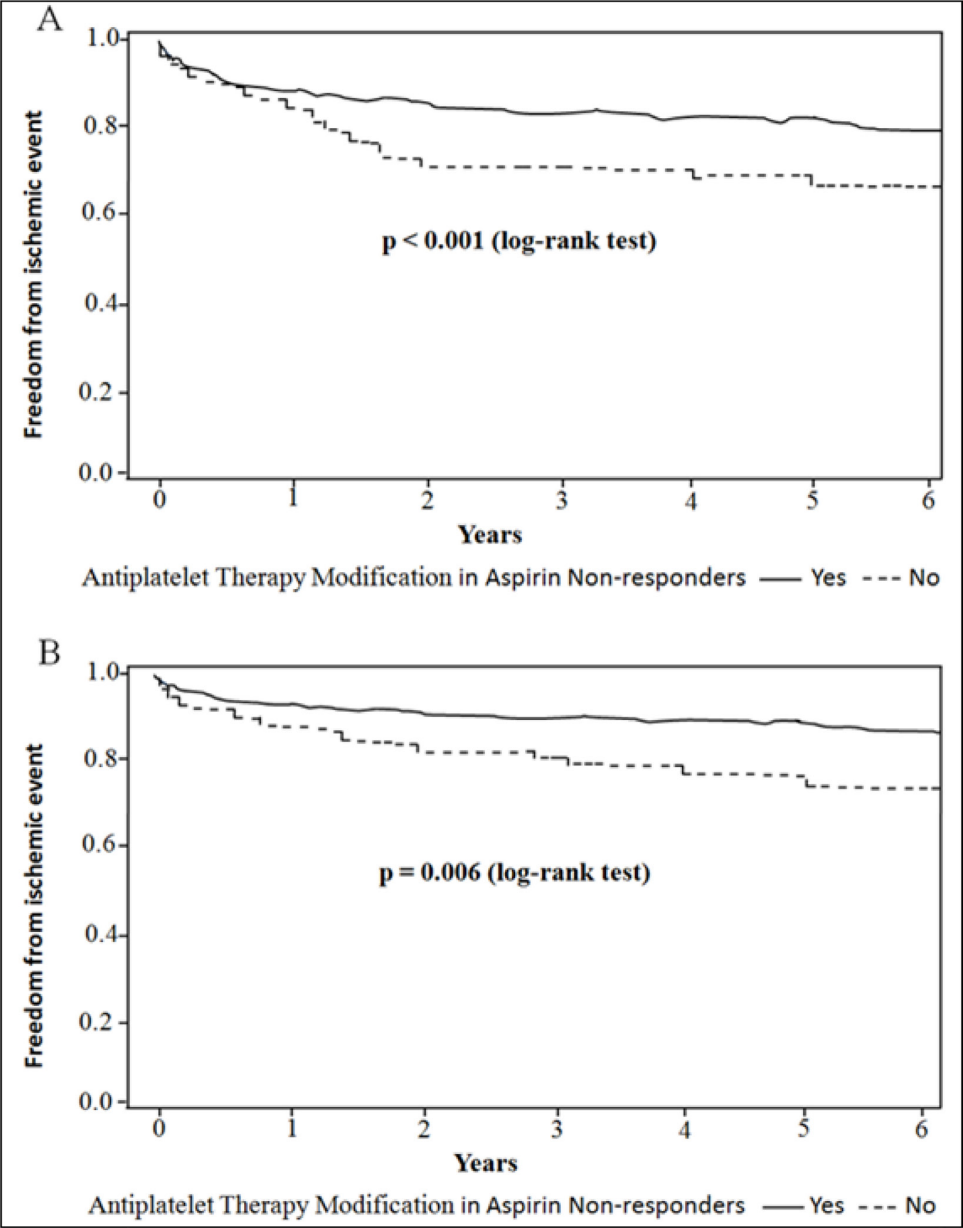
Kaplan-Maier analysis of clinical outcomes associated with and without modifying antiplatelet therapy after platelet function testing in aspirin non-responders Freedom from (**A**) ischemic event; (**B**) ischemic stroke are compared between patients with and without antiplatelet therapy modification with a log-rank test and its associated *P* value.

In patients who were nonresponsive to aspirin (*n =* 223), ischemic events occurred in 51 (22.9%) patients (35 had ischemic stroke, 9 had TIA and 7 had MI). There were no significant differences in baseline characteristics between the patients with (*n =* 154) and without (*n =* 69) ATM for the aspirin nonresponse subgroup (Table 5). However, the patients with ATM compared with the patients without modification were associated with decreased ischemic events (18.2% versus 33.3%, *P* = 0.02, Table 5), which was primarily due to a decrease in ischemic stroke (11.7% versus 24.6%, *P* = 0.008, Table 5). Kaplan-Meier estimates of cumulative freedom from ischemic event (log-rank *P* < 0.001, Figure 1A), and ischemic stroke (log-rank *P* = 0.006, Figure 1B) were significantly lower in patients without ATM compared with patients who underwent modification in aspirin non-responders. However, there were no significant differences in incidence rates of bleeding events and death between the two groups (Table 5).

**Table 5:** Baseline characteristics and clinical outcomes in aspirin non-responders

Variable	Antiplatelet Therapy Modification		*P* value
	Yes (*n* = 154)	No (*n* = 69)	
Age (years)	70.9 ± 11.9	70.4 ± 10.8	0.76
Men (*n*, %)	70 (45.5)	37 (53.6)	0.27
Diabetes mellitus (*n*, %)	54 (35.4)	25 (36.2)	0.91
Hypertension (*n*, %)	116 (75.3)	50 (72.5)	0.67
Current smoking (*n*, %)	46 (29.9)	17 (24.6)	0.43
Previous MI (n, %)	4 (2.6)	2 (2.9)	0.99
NIHSS score at enrollment	5.8 ± 2.3	5.9 ± 2.1	0.65
Hyperlipidemia (*n*, %)	127 (82.5)	61 (88.4)	0.26
Fasting glucose (mmol/L)	7.1 ± 2.2	7.3 ± 2.4	0.56
Platelet count (×10^9^/L)	189.2 ± 25.8	195.2 ± 29.2	0.14
Stroke subtype Atherothrombotic (*n*, %) Small artery disease (*n*, %)	95 (61.7) 59 (38.3)	44 (63.8) 25 (36.2)	0.78 0.78
Ischemic events (*n*, %)	28 (18.2)	23 (33.3)	0.02
Ischemic stroke (*n*, %)	18 (11.7)	17 (24.6)	0.008
Transient ischemic attack (*n*, %)	6 (3.9)	3 (4.3)	0.92
MI (*n*, %)	4 (2.6)	3 (4.3)	0.48
Any bleeding event	15 (9.7)	6 (8.7)	0.81
GUSTO minor (*n*, %)	9 (5.8)	4 (5.8)	0.99
GUSTO moderate (*n*, %)	4 (2.6)	1 (1.4)	0.64
GUSTO severe (*n*, %) Gastrointestinal bleeding (*n*, %) Intracerebral hemorrhage (*n*, %)	2 (1.3) 9 (5.8) 1 (0.6)	1 (1.4) 3 (4.3) 1 (1.4)	0.99 0.99 0.52
Death (*n*, %)	4 (2.6)	3 (4.3)	0.67

MI, myocardial infarction; NIHSS, National Institutes of Health Stroke Scale; GUSTO, Global Use of Strategies to Open Occluded Coronary Arteries.

In patients with AS (*n =* 589), ischemic events occurred in 130 (22.1%) patients (85 had ischemic stroke, 28 had TIA and 17 had MI). There were no significant differences in clinical outcomes between the AS patients with (*n =* 50) and without (*n =* 539) ATM (all *P* > 0.05, Supplementary Table 2).

With regard to aspirin non-response patients and AS patients who did not receive ATM (*n =* 69 and *n =* 539, respectively), the incidence rates of ischemic events and ischemic stroke in patients with aspirin non-response were significantly higher than patients with AS (*P* = 0.03 and *P* = 0.02, respectively, Table 6). However, there were no significant differences in incidence rates of bleeding events and death between the two groups (Table 6).

**Table 6:** Clinical Outcomes between aspirin non-response patients and aspirin sensitive patients who did not receive the modified antiplatelet therapy

Variable	aspirin sensitive patients (*n* = 539)	aspirin non-responders (*n* = 69)	*P* value
Ischemic events (*n*, %)	115 (21.3)	23 (33.3)	0.03
Ischemic stroke (*n*, %)	74 (13.7)	17 (24.6)	0.02
Transient ischemic attack (*n*, %)	26 (4.8)	3 (4.3)	0.88
Myocardial infarction (*n*, %)	15 (3.3)	3 (4.3)	0.43
Any bleeding event	54 (10.0)	6 (8.7)	0.74
GUSTO minor (*n*, %)	27 (5.0)	4 (5.8)	0.81
GUSTO moderate (*n*, %)	20 (3.7)	1 (1.4)	0.36
GUSTO severe (*n*, %) Gastrointestinal bleeding (*n*, %) Intracerebral hemorrhage (*n*, %)	7 (1.3) 38 (7.1) 6 (1.1)	1 (1.4) 3 (4.3) 1 (1.4)	0.98 0.45 0.86
Death (*n*, %)	16 (3.0)	3 (4.3)	0.56

GUSTO, Global Use of Strategies to Open Occluded Coronary Arteries.

In patients with aspirin nonresponse, the unadjusted and propensity score-adjusted hazard ratios for clinical outcomes with and without modification of antiplatelet therapy are shown in Table 7. With propensity score adjustment, ATM was associated with lower rates of ischemic event (hazard ratio, 0.67; 95% confidence interval (CI), 0.62–0.97; *P* = 0.01) or ischemic stroke (hazard ratio, 0.70; 95% CI, 0.63–0.98; *P* = 0.03) compared with no modification. No significant differences were seen in the propensity score-adjusted individual rates of death, or bleeding events between the two groups. In additional analyses performed after propensity score matching of patients in the ATM (*n* = 72) and no modification (*n* = 72) groups, rates of ischemic event and ischemic stroke remained significantly lower in the ATM group (hazard ratio, 0.69; 95% CI, 0.57–0.94; *P* = 0.02, and hazard ratio, 0.67; 95% CI, 0.62–0.97; *P* = 0.03, respectively). No significant difference in death, or bleeding events was seen between the 2 matched groups.

**Table 7:** Unadjusted and adjusted hazards ratios (HRs) for clinical outcomes with and without antiplatelet therapy modification in aspirin non-responders

Clinical Outcome	Unadjusted		Propensity Score Adjusted		Propensity Score Matched	
	HR (95% CI)	*P* value	HR (95% CI)	*P* value	HR (95% CI)	*P* value
Ichemic events	0.63 (0.51–0.88)	<0.001	0.67 (0.62–0.97)	0.01	0.69 (0.57–0.94)	0.02
Ischemic stroke	0.66 (0.56–0.98)	0.004	0.70 (0.63–0.98)	0.03	0.67 (0.62–0.97)	0.03
Bleeding event	1.33 (0.78–4.38)	0.61	1.39 (0.89–4.45)	0.69	1.25 (0.87–4.85)	0.17
Death	1.35 (0.64–3.85)	0.65	1.41 (0.91–4.07)	0.64	1.16 (0.88–4.29)	0.41

## DISCUSSION

In present study, all patients underwent platelet function testing, antiplatelet therapy was modified in 204 patients after platelet function testing. The incidence rates of ischemic events, death, bleeding events were not significantly different between the patients who underwent ATM compared with no modification. However, in patients with aspirin nonresponse, antiplatelet therapy modification was associated with decreased ischemic events and ischemic stroke compared with no modification.

The prevalence of aspirin nonresponse was 27.5% in present study, and was similar to the prevalence reported in our previous studies [8, 11, 21] and some other studies [9, 22]. A recent systematic review and meta-analysis showed that the prevalence of high on-treatment of platelet reactivity (HTPR) on aspirin was 23% (95% CI: 20–28%), and the patients with HTPR had a significantly higher risk for ischemic stroke recurrence (relative risk = 1.81, 95% CI: 1.30–2.52; *P* < 0.001) [23]. The finding is consistent with our present study. The mechanisms associated with aspirin nonresponse are complex and mutilfactorial, such as noncompliance, diabetes mellitus, reduced absorption, the biosynthesis of thromboxane A2 from pathways not inhibited by aspirin as well as alternative pathways involved in platelet activation not blocked by aspirin [9, 11, 21, 22]. In present study, our results also showed that the diabetes mellitus was associated with aspirin nonresponse. Patients with diabetes mellitus were usually associated with hyperlipidemia, chronic inflammation, platelet dysfunction and endothelial dysfunction, and these result in poor responsiveness to aspirin [24]. Other potential mechanisms for aspirin nonresponse in patients with diabetes mellitus may include increased circulating ADP, calcium, or esterase levels, as well as platelet turnover, the expression of P2Y12 receptors, or the upregulation of other platelet activation pathways [25]. Thus, intensive antiplatelet therapy may be important in diabetic patients sustaining an ischemic stroke.

Several studies have shown that nonresponse to aspirin is associated with more frequent neurologic deterioration, less frequent clinical improvement, and greater risk of recurrent ischemic events in patients with acute ischemic stroke [8, 11, 26, 27]. Alberts *et al* suggested that modification in antiplatelet therapy according to platelet function testing was reasonable [20]. However, there are some debates about the clinical efficacy and safety of platelet function-guided modification in antiplatelet therapy in patients with acute ischemic stroke. Our current results showed that the incidence rates of ischemic events, death, bleeding events were not significantly different between the patients with and without ATM after ischemic stroke, and were consistent with previous studies [14, 17]. The majority of aspirin nonresponse reported in the literature may be the result of poor adherence and clinical factors that predict aspirin nonresponse are not consistent between different platelet function tests [28]. Thus, platelet function testing is not recommended in the current guidelines for management of ischemic stroke [5].

Nevertheless, key observation were identified in the present study via stratified analyses, stratified analyses revealed that the incidence rates of ischemic events and ischemic stroke were significantly higher in patients with aspirin non-response than patients with AS who did not receive ATM, and ATM was associated with decreasing ischemic events and ischemic stroke compared with no modification in patients with aspirin nonresponse. However, our findings were inconsistent with other results [14, 17]. Collet *et al* [17] reported that there were no significant improvements in clinical outcomes with platelet-function monitoring and treatment adjustment for coronary stenting. Depta *et al* [14] showed that modification in antiplatelet therapy after an ischemic stroke or TIA was associated with significantly increased rates of death, ischemic events, or bleeding compared with no modification. However, only 324 patients with ischemic stroke or TIA were enrolled in the retrospective study, the small samples is may be a important cause for the conflicting results.

In patients with aspirin nonresponse, preventing recurrent ischemic stroke after ischemic stroke with aspirin therapy remains a challenge. There are no standardized treatment recommendations for these patients with aspirin nonresponse. In present study, ATM included changing from aspirin to clopidogrel or cilostazol (*n =* 149), adding clopidogrel to aspirin (*n =* 37), and increasing the aspirin dosage (*n =* 18). There were no significant differences in incidence rates of ischemic events and death among the different ATM. However, the rate of bleeding was significantly higher in patients with modification of adding clopidogrel to aspirin or increasing the aspirin dosage than patients with modifications of changing from aspirin to clopidogrel or cilostazol, or without modification. Increasing the dose of aspirin might reduce the incidence of aspirin nonresponse, and prevent occurrence of vascular events [12, 13], but higher doses of aspirin may increase the risk of a hemorrhagic event [14]. Dual antiplatelet therapy with aspirin and clopidogrel for the first 21 days or 30 days in patients with acute ischemic stroke can reduce the risk of stroke, and improve 6-month outcome [15, 29, 30]. However, long-term combination of clopidogrel and aspirin was not more effective than clopidogrel alone in preventing recurrent ischemic events, and the risk of life-threatening or major bleeding is increased [16]. Our results also showed the rate of bleeding was higher in patients in whom clopidogrel was added or dose of aspirin was increased than patients without modification. Thus, increasing the dose of aspirin or long-term dual antiplatelet therapy with aspirin and clopidogrel for the secondary prevention of ischemic stroke were inadequate for these patients. Substitution of aspirin with another antiplatelet drug (like clopidogrel or cilostazol) is thought to optimize regime, and may help prevent the occurrence of vascular events [31, 32]. The Clopidogrel versus Aspirin in Patients at Risk of Ischemic Events (CAPRIE) trial demonstrated that clopidogrel is more effective than aspirin in reducing the combined risk of ischemic stroke, MI, or vascular death in patients with atherosclerotic vascular disease [33]. A meta-analysis to estimate the efficacy of antiplatelet agents for secondary prevention of recurrent stroke demonstrated that cilostazol was significantly more efficient than other antiplatelet agents in Asian patients [32]. These were consistent with our current findings. However, further randomized-controlled trials are needed to validate our findings.

The risk of recurrent stroke is very high after ischemic stroke, and aspirin is recommended to reduce the risk of recurrent ischemic events in patients with ischemic stroke. However, the response to aspirin is variable. Our previous studies showed that nonresponse to aspirin in patients with ischemic stroke is associated with an increased risk of recurrence ischemic stroke and worse functional status, and platelet function testing may be useful as a marker of increased risk for recurrent events after ischemic stroke [8,11]. In presents study, our results revealed that ATM after platelet function testing was associated with decreasing ischemic events and ischemic stroke in patients with aspirin nonresponse. Up to date, few studies assessed the efficacy and safety of modifications in antiplatelet therapy according to platelet function testing in patients with acute ischemic stroke. The results of our study indicate that platelet function testing may be useful to guide ATM and optimize clinical outcomes in patients with aspirin nonresponse. Our these findings could be useful to guide the precise treatment of antiplatelet drugs, decrease the risk of recurrent ischemic events, improve functional status, and develop more effective drugs to prevent recurrent ischemic events after ischemic stroke.

Several important limitations of our study should be considered. First, our study is retrospective and observational, and this may limit the generalizability of the results. Additionally, the diverse modifications in antiplatelet regimens used after platelet function testing were at the physician’s discretion. It is unknown what clinical factors led each physician to decide which therapeutic regimen to use after platelet function testing, thus making it very difficult to control for selection bias. Second, several laboratory tests are used to assess the response to aspirin, including LTA, bleeding time, platelet function analyzer-100, the VerifyNow Aspirin system. Each method has its own advantages and disadvantages [33]. However, platelet aggregation was only measured using the LTA in this study. Third, retesting platelet function after antiplatelet therapy modification was only performed in 105 patients, the infrequency of retesting limited our ability to determine if responsiveness after antiplatelet therapy modification resulted in any clinical benefit. Fourth, although careful analysis was performed to account for any differences between patients with and without antiplatelet therapy modification, unknown confounders may have contributed to the differences in clinical outcomes between both groups. Furthermore, the current study may also have possible bias due to the three-center, relative small sample size. Therefore, our findings must be validated in multi-center, larger sample size, and randomized-controlled trials.

## CONCLUSIONS

In patients with aspirin nonresponse, antiplatelet therapy modification was associated with decreased ischemic events and ischemic stroke compared with no modification. The results indicate that platelet function testing is may be useful to guide antiplatelet therapy modification, and optimize clinical outcomes, although our results should be interpreted with caution given the possible confounding role of selection bias. Randomized-controlled trials are needed to determine if a platelet function-guided approach is beneficial and safe to prevent recurrent events after ischemic stroke in future.

## MATERIALS AND METHODS

### Study population

This retrospective, multi-centre study was jointly conducted by the People’s Hospital of Deyang City, the second, and third Affiliated Hospital of Wenzhou Medical University. The study protocol was approved by the Ethics Committee at the participating hospitals. Written informed consent was obtained from each patient. Institutional Review Board approval was obtained on January 31, 2016.

We consecutively enrolled patients who underwent a first-ever ischemic stroke and were admitted to the participating hospitals within 72 h of the onset of stroke between August 2010 and December 2014. The inclusion criteria were: (1) age ≥ 40 years old; (2) all patients underwent platelet function testing; (3) all patients were receiving aspirin monotherapy before the platelet function testing; (4) absence of endovascular or surgical treatment for stroke. Exclusion criteria were: (1) cerebral embolism or undetermined etiologies of ischemic stroke; (2) patients whose antiplatelet therapy was decreased or who had warfarin added during observational phase; (3) loss to follow-up. A total of 883 patients met above inclusion criteria and exclusion criteria. However, 71 patients declined to participate this study. Thus, 812 patients were enrolled. The overall response rate was approximately 92% (812/883) [92.6% (287/310) in the People’s Hospital of Deyang City, 91.2% (207/227) and 91.9% (318/346) in the second and third Affiliated Hospital of Wenzhou Medical University, respectively].

All enrolled patients received standard therapies based on the guidelines for the prevention of stroke in patients with stroke and TIA [5]. All patient’s data were obtained through the electronic medical record system and/or paper charts and were independently verified by the authors. Hypertension was defined as the mean of three independent measures of BP ≥ 140/90 mmHg or the use of antihypertensive drugs. Diabetes mellitus was diagnosed by any one or a combination of fasting glucose level > 7.8 mmol/L, > 11.1 mmol/L 2 h after oral glucose challenge, and use of hypoglycemic drugs. Dyslipidemia was defined as TC > 200 mg/dL, TG > 180 mg/dL or use of lipid-lowering medication. Cigarette smoking was defined as smoking of at least one cigarette per day for more than 1 year [34].

### Platelet function testing and definition of antiplatelet resistance

Blood samples were collected at 7–10 days after aspirin therapy. Platelet function was measured by light transmittance aggregometry (LTA). The procedures and consistency tests were performed as described in our previous studies [8, 10, 11]. In the present study, aspirin resistance (AR) was defined as a mean platelet aggregation ≥20% with 0.5 mM arachidonic acid (AA) and ≥70% with 10 μM adenosine diphosphate (ADP) at 7–10 days after aspirin therapy. Patients who meet only 1 of the above 2 criteria are defined as aspirin semi-resistance. For the purposes of our study, aspirin non-response was defined as any patient meeting either criteria and currently on aspirin [11]. Otherwise, patients were considered aspirin sensitive (AS).

### Definition of antiplatelet therapy modification

The definition used for antiplatelet therapy modification (ATM) was any change in the patient’s antiplatelet regimen within 24 hours after the platelet function testing result was made available. Change in antiplatelet therapy was defined as any increasing the dosage of aspirin (200 mg/d increase to 300 mg/d), adding an additional antiplatelet agent to aspirin (add clopidogrel to aspirin), or switching to a more potent antiplatelet agent (eg, change from aspirin to clopidogrel or cilostazol). The following patients needed to receive ATM: (1) aspirin non-responders, and these non-responders were willing to receive ATM; (2) side effects of aspirin, such as allergic to aspirin, gastrointestinal bleeding, skin or mucosal bleeding, and severe nausea and vomiting. For the patients with side effects of aspirin, aspirin was switched to clopidogrel or cilostazol. For aspirin non-responders, one following ATM was selected: (1) increasing the dosage of aspirin; (2) adding an additional antiplatelet agent to aspirin; (3) switching to a more potent antiplatelet agent. This was a multi-center, retrospective study, not a randomized-controlled trial. In addition, up to date, there are no standardized treatment recommendations for the aspirin non-responders. Thus, the diverse modifications in antiplatelet regimens used after platelet function testing were at the discretion of the treating physician in this study.

### Assessment of clinical outcomes

The primary outcome of the study was ischemic events. Ischemic events were defined as an ischemic stroke, TIA, myocardial infarction (MI). Ischemic stroke was defined as any non-hemorrhagic or embolic stroke with loss of neurological function caused by an ischemic event with residual symptoms at least 24 hours after onset, where as TIA was defined as loss of neurological function without residual deficit at 24 hours. MI was defined as the presence of at least two of these criteria: prolonged angina >30 min; total creatinine kinase isoenzyme elevation more than twice the upper limit of normal; electrocardiographic evidence of infarction.

Secondary outcomes included death and bleeding events. Death was defined as all-cause mortality. Bleeding events were defined according to the Global Use of Strategies to Open Occluded Coronary Arteries (GUSTO) bleeding classification [35]. GUSTO Severe or life-threatening bleeding was defined as any intracranial hemorrhage or bleeding that causes hemodynamic compromise requiring intervention. Any bleeding that required blood transfusion in the absence of hemodynamic compromise was considered GUSTO moderate bleeding. GUSTO minor bleeding was defined as any bleeding that did not meet criteria for severe or moderate bleeding.

Follow-up was performed by telephone interview and by reviewing the medical charts of each participant regardless of aspirin resistance status. The researchers who performed follow-up interviews were blinded to aspirin sensitivity status. Scheduled follow-up telephone calls were made after discharge to support proper compliance, answer any queries, and record complaints of any side effects. For those patients who reached at least one of the primary end points, a medical chart review was initiated to determine whether the event met the definitions described earlier. The terminal time of follow-up was December 31, 2016.

### Statistical analysis

All statistical analyses were performed using SPSS 16.0 (SPSS Inc., Chicago, IL, USA). Differences between the antiplatelet therapy modification and no modification groups were analyzed by univariate methods. Categorical variables are presented as frequencies and percentages and compared using the Chi-square or Fisher’s exact tests. Continuous variables are expressed as mean ± Standard Deviation (SD) and compared using the Student’s *t*-test. Survival function estimates for clinical outcomes were evaluated through Kaplan-Meier analyses. Survival curves were truncated at year 5. The log-rank test was used to identify differences between antiplatelet therapy modification and no modification groups.

Propensity scores were created for antiplatelet therapy modification and no modification groups based on patient characteristics. The following variables were used to calculate the propensity score: age, male, inpatient, smoking status, diabetes mellitus, hypertension, hyperlipidemia, prior MI, prior percutaneous coronary intervention, prior coronary artery bypass graft(s), history of aspirin, antihypertensive drugs, hypoglycemic drugs, and statins. Covariate balance between groups was evaluated by examining the Wald chi-square statistic before and after propensity score adjustment. After adjusting for propensity score, none of the variables used to create propensity score were found to be significantly different between groups. An additional analysis on matched propensity scores was conducted and standardized differences were calculated to determine covariate balance before and after matching. A Cox proportional hazards model for each outcome was created with and without propensity score adjustment. All tests were two-sided, and *P* values of 0.05 were considered to represent statistical significance.

## SUPPLEMENTARY MATERIALS TABLES



## Abbreviations

CI: confidence interval
TIA: transient ischemic attack
RIS: recurrence ischemic stroke
TC: total cholesterol
TG: triglycerides
LTA: light transmittance aggregometry
AR: aspirin resistance
AS: aspirin sensitive
AA: arachidonic acid
ADP: adenosine diphosphate
GUSTO: Global Use of Strategies to Open Occluded Coronary Arteries
MI: myocardial infarction
HTPR: high on-treatment of platelet reactivity
NIHSS: National Institutes of Health Stroke Scale
