# Supplementary material for: Platelet function-guided modification in antiplatelet therapy after acute ischemic stroke is associated with clinical outcomes in patients with aspirin nonresponse
Source: Oncotarget. 2017 Nov 7;8(63):106258–69. doi: 10.18632/oncotarget.22293 (PMC5739731; doi:10.18632/oncotarget.22293)
Supplement: Supplementary file 1 [file oncotarget-08-106258-s001.pdf]

Platelet function-guided modification in antiplatelet therapy after acute ischemic stroke is associated with clinical outcomes in patients with aspirin nonresponse

SUPPLEMENTARY MATERIALS

Supplementary Table 1: Clinical outcomes in different antiplatelet therapy modification

Variable	changing aspirin (*n* = 149)	adding clopidogrel (*n* = 37)	increasing dose of aspirin (*n* = 18)	*P* value
Ischemic events (*n*, %)	31 (20.8)	8 (21.6)	4 (22.2)	0.97
Ischemic stroke (*n*, %)	21 (14.1)	5 (13.5)	3 (16.7)	0.96
Transient ischemic attack (*n*, %)	5 (3.4)	2 (5.4)	1 (5.6)	0.81
Myocardial infarction (*n*, %)	5 (3.4)	1 (2.7)	0 (0.0)	0.59
Any bleeding event	9 (6.0)	10 (27.0)	4 (22.2)	<0.001
GUSTO minor (*n*, %)	3 (2.0)	7 (18.9)	2 (11.1)	<0.001
GUSTO moderate (*n*, %)	4 (2.7)	2 (5.4)	2 (11.1)	0.17
GUSTO severe (*n*, %)	2 (1.3)	1 (2.7)	0 (0.0)	0.71
Gastrointestinal bleeding (*n*, %)	7 (4.7)	3 (8.1)	2 (11.1)	0.93
Intracerebral hemorrhage (*n*, %)	1 (0.7)	1 (2.7)	0 (0.0)	0.34
Death (*n*, %)	4 (2.7)	2 (5.4)	1 (5.6)	0.64

GUSTO, Global Use of Strategies to Open Occluded Coronary Arteries

Supplementary Table 2: Clinical outcomes in aspirin sensitive patients

Variable	Antiplatelet Therapy Modification		*P* value
	No (*n* = 539)	Yes (*n* = 50)	
Ischemic events (*n*, %)	115 (21.3)	15 (3.0)	0.18
Ischemic stroke (*n*, %)	74 (13.7)	11 (22.0)	0.12
Transient ischemic attack (*n*, %)	26 (4.8)	2 (4.0)	0.81
Myocardial infarction (*n*, %)	15 (3.3)	2 (4.0)	0.82
Any bleeding event	54 (10.0)	8 (16.0)	0.21
GUSTO minor (*n*, %)	27 (5.0)	3 (6.0)	0.76
GUSTO moderate (*n*, %)	20 (3.7)	4 (8.0)	0.13
GUSTO severe (*n*, %)	7 (1.3)	1 (2.0)	0.68
Gastrointestinal bleeding (*n*, %)	38 (7.1)	3 (6.0)	0.79
Intracerebral hemorrhage (*n*, %)	6 (1.1)	1 (2.0)	0.54
Death (*n*, %)	16 (3.0)	3 (6.0)	0.24

GUSTO, Global Use of Strategies to Open Occluded Coronary Arteries
